# Macronutrient intake modulates impact of *EcoRI* polymorphism of ApoB gene on lipid profile and inflammatory markers in patients with type 2 diabetes

**DOI:** 10.1038/s41598-022-13330-x

**Published:** 2022-06-22

**Authors:** Faezeh Abaj, Fariba Koohdani

**Affiliations:** 1 Department of Community Nutrition, School of Nutritional Sciences and Dietetics, Tehran University of Medical Sciences, Tehran, Iran; 2 Department of Cellular and Molecular Nutrition, School of Nutritional Sciences and Dietetics, Tehran University of Medical Sciences (TUMS), Tehran, PO Box: 141556117, Iran

**Keywords:** Genetic association study, Genetic interaction, Medical genetics, Metabolic disorders, Dyslipidaemias

## Abstract

We sought to examine whether dietary intakes may affect the relationship between ApoB EcoRI and lipid profile, as well as serum inflammatory markers, in patients with type 2 diabetes (T2DM). This current study consisted of 648 diabetic patients. Dietary intake was calculated by a food frequency questionnaire. Biochemical markers (high-density lipoprotein (HDL), total cholesterol (TC), LDL, TG, CRP, IL-18, PGF2α) were measured based on standard protocols. Genotyping of the Apo-B polymorphisms (rs1042031) was conducted by the PCR–RFLP method. The gene-diet interactions were evaluated using GLMs. In comparison to GG homozygotes, A-allele carriers with above the median -CHO intake (≥ 54 percent of total energy) had considerably greater TC and PGF2a concentrations. Furthermore, as compared to GG homozygotes, A-allele carriers with above the median protein intake (≥ 14 percent of total energy) had higher serum levels of TG (*P* = 0.001), CRP (*P* = 0.02), TG/HDL (*P* = 0.005), and LDL/HDL (*P* = 0.04) ratios. Moreover, A-allele carriers with above the median total fat intake (≥ 35 percent of total calories) had significantly higher TC level (*P* = 0.04) and LDL/HDL (*P* = 0.04) ratios compared to GG homozygotes. Furthermore, when compared to GG homozygotes, A-allele carriers who consumed above the median cholesterol (> 196 mg) had greater TG (*P* = 0.04), TG/HDL (*P* = 0.01) ratio, and IL-18 (*P* = 0.02). Furthermore, diabetic patients with the GA, AA genotype who consume above the median cholesterol had lower ghrelin levels (*P* = 0.01). In terms of LDL/HDL ratio, ApoB EcoRI and dietary intakes of specific fatty acids (≥ 9 percent for SFA and ≥ 12 percent for MUFA) had significant interaction. LDL/HDL ratio is greater in A-allele carriers with above the median SFA intake (*P* = 0.04), also when they consumed above the median MUFA this association was inverse (*P* = 0.04). Our study showed that plasma lipid levels in participants carrying the (AA or AG) genotype were found to be more responsive to increasing the percentage of energy derived from dietary fat, CHO, protein, SFA, and cholesterol consumption. Therefore, patients with a higher genetic susceptibility (AA or AG) seemed to have greater metabolic markers with a higher percentage of macronutrient consumption. Also, ApoB EcoRI correlations with metabolic markers might be attenuated with above the median MUFA consumption.

## Introduction

Type 2 diabetes mellitus (T2DM) is now one of the most common non-communicable diseases in the world. This is due to the fact that T2DM is recognized as one of the leading causes of death worldwide^1^. According to current trends, the number of fatalities caused by T2DM is expected to rise from 285 million adults in 2012 to over 439 million individuals by 2030^2,3^. In diabetes mellitus, dyslipidemia is one of the most important risk factors for cardiovascular disease^4^. Indeed, T2DM is correlated to altered lipid profiles, including raised TG and/or low HDL, a condition known as metabolic dyslipidemia^5,6^.

Findings indicate that genetic differences have a key role in the prevention and treatment of a variety of chronic diseases, including diabetes and dyslipidemia^7–10^. Genetic variables are known to be independent predictors for both dyslipidemia and diabetes^11,12^. As a result, it is critical to find the genes that cause T2DM-related metabolic problems and to develop novel treatments^13,14^. Particularly, the gene encoding APOB is one of the most widely anticipated genes that could alter lipid metabolism in T2DM^15^. The Apo-B polymorphism EcoRI (rs1042031) has been found to alter total and LDL cholesterol levels^16,17^.

Diet plays a significant impact in the initiation and progression of T2DM-related chronic diseases^18,19^. Additionally, some studies have found an association between dietary macronutrient intake and metabolic diseases such as dyslipidemia and inflammatory indicators^20,21^. Several recent investigations have found evidence that the response of plasma lipid levels to dietary changes is influenced by genetic factors^22^.

According to previous research, the ApoB EcoRI (R-) variant reduces the variation in total cholesterol levels between identical twins. Because identical twins had the same genetic code, differences in cholesterol levels between co-twins have to be attributable to a variety of circumstances, including dietary differences^23–25^. Interestingly, multiple studies have suggested that EcoRI genetic diversity interacts with other risk variables such as dietary fatty acid and cholesterol intake, indicating a positive relationship between EcoRI and hypercholesterolemia^26^.

To the best of the authors' knowledge, there has been no work thus far aimed at evaluating the interaction between EcoRI rs1042031 variant and macronutrient intake on serum levels of lipid and inflammatory markers, leptin and ghrelin. The objectives of this study were to evaluate the association between metabolic markers and EcoRI polymorphism and identify the interaction of EcoRI rs1042031 with the types of dietary macronutrient intake, in regards of metabolic risk factor in T2DM patients.

## Methods and materials

### Study population

648 patients (252 men and 396 women) were selected for this cross-sectional study from Tehran's referral Diabetes Centers. The present study established in 2019, and the information includes dietary intakes and genetic data collected in previous studies^27^. Subjects with fasting blood glucose (FBG) ≥ 126 mg/dl, taking diabetic medicines, or subjects fulfilling both criteria were included. Pregnant and lactating women, patients being treated with insulin, and drug-addicted subjects were excluded. The interview-based evaluations were also used to collect general information such as age, gender, work and education status, smoking and alcohol behaviors, lipid-lowering medicine use, T2DM duration, and family history of the disease. All methods were performed in accordance with the Declaration of Helsinki.

### Anthropometric and physical activity assessments

Anthropometric values were measured, including weight without shoes on a digital scale with 0.1-kg reliability and height without shoes on 0.1-cm-accuracy height gauges. Weight (kg)/height2 was used to determine the body mass index (BMI) (m). The Tehran University of Medical Sciences Ethics Committee approved the study, and all patients signed an informed consent form before being enrolled. The International Physical Activity Questionnaire (IPAQ) modified version was used to assess physical activity. The IPAQ's reliability and validity have previously been tested among Iranian adults^28^.

### Laboratory tests

All blood samples were obtained at TUMS' nutrition laboratory after 12–14 h of fasting, centrifuged, and stored at − 80 °C. Enzymatic methods were used to quantify serum lipid biomarkers such as HDL, TC, LDL, and TG (Pars Azmun Co., Tehran, Iran). The levels of leptin and ghrelin in the plasma were also determined by the ELISA method (Bioassay Technology Co, China, and Mediagnost, Germany, respectively). Inflammatory markers (CRP, IL-18, and PGF2) were evaluated according to the manufacturer's instructions using enzyme-linked immunosorbent assay kits (R&D Systems, Techne Corporation, Minneapolis, MN).

### Genotyping

The salting-out procedure was used to extract genomic DNA. The polymerase chain reaction was used to determine genotyping (PCR–RFLP). The following primers were used to amplify rs1042031: (forward primer: CACTGGGACCTACCAAGAG; reverse primer: CACTGGGACCTACCAAGAG) CTCGAAAGGAAGTGTAATCAC (reverse primer). The reaction included 35 cycles of denaturation at 94 °C for three minutes, followed by 35 cycles of 30 s at 94 °C, 30 s at 56 °C, 40 s at 72 °C, and one cycle of seven minutes at 72 °C and five minutes at 4 °C. PCR-Thermocycler was used to perform the PCR reaction (PEQLAB; GmbH96; Germany).

### Dietary assessments

Expert dietitians evaluated dietary intakes using a 147-item, semi-quantitative FFQ^29^. The frequency of each food item consumed over the previous year was reported by the participants and calculated to grams per day using home measurements. The Iranian Food Composition Table (FCT) and N4 software were used to calculate total energy and dietary elements. The nutritional density approach was also used to adjust this dietary intake for total energy intake. In our study, dietary intakes were categorized into two groups according to the median value, below and above or equal based on our pervious study^27^.The carbohydrate, protein, total fat, saturated fatty acid, MUFA and cholesterol were categorized into two groups, according to their medians of the population (54, 14, 35, 9,12 percent and 196 mg, respectively).

### Statistical analysis

Initially, the Kolmogorov–Smirnov test was applied to determine distribution normality. The 2 test was used to analyze the Hardy–Weinberg equilibrium and categorical variable comparison. Independent Samples T-tests were used to compare quantitative variables between genotypes. The interaction between EcoRI polymorphisms and macronutrient intake on lipid profile, as well as serum inflammatory markers were verified by Generalized Linear Models (GLZM). We used dietary intake groups and genotype categories as a fixed factor and cofounding factors as a covariate then we made an interaction model. The GG genotype has no risk allele and is used as a reference. Moreover, the intake of macronutrients below the median is used as a reference. Biochemical markers (high-density lipoprotein (HDL), total cholesterol (TC), LDL, TG, CRP, IL-18, PGF2α), were response variables, while EcoRI genotypes and each macronutrient intake were considered as factor variables. Model 1: unadjusted; Model 2: adjusted for age, gender, physical activity, alcohol consumption and lipid-lowering medications; Model 3: adjusted for variables in model 2 plus for smoking, total energy intakes and fiber intake. All data were statistically analyzed using SPSS (version 25; SPSS Inc., IL). *P *value < 0.05 was used to determine statistical significance.

## Result

A total of 648 Iranian diabetic patients were categorized based on rs1042031 genotypes and divided into two groups: GA, AA genotypes (n = 145), GG genotype (n = 503). Genotypic and allelic frequencies of *EcoRI* in men and women are presented in Table 1. Besides, Genotype frequency for dominant homozygote (GG), heterozygote (GA), and recessive homozygote (AA) was 77.7%, 21.1%, and 1.2%, respectively have shown in Table 1. The genotype frequency (*P* = 0.23) shows no divergence from Hardy–Weinberg Equilibrium (HWE). Furthermore, there was no significant correlation found between the ApoB EcoRI polymorphism and inflammatory markers or dietary intake (*P* > 0.05). Diabetic patients with GA, AA genotype had more BMI (*P* = 0.004) and WC (*P* = 0. 004) compared to GG homozygotes. Furthermore, we revealed a correlation between the ApoB EcoRI polymorphism and serum HDL-C concentration. As a result, plasma HDL-C levels in A-allele carriers were considerably greater than in GG homozygotes. Also, no correlation was found between this polymorphism and other lipid variables (*P* ≥ 0.05) (Table 2).

**Table 1:** Distribution of Genotype and allele frequencies of EcoR1 polymorphism of the Apo B gene in patients with type 2 diabetes mellitus.

Gender	Genotype				Relative allele	
	GG	GA	AA	Total	G	A
Male	205 (81.3)	44 (17.5)	3 (1.2)	252 (100)	454 (39.7)	60 (36.8)
Female	298 (75.3)	93 (23.5)	5 (1.3)	396 (100)	689 (60.3)	103 (63.2)
Total	503 (77.7%)	137 (21.1%)	8 (1.2%)	648 (100)	1143 (100.0)	163 (100.0)

Values are frequency (percent).

**Table 2:** General, anthropometrical and dietary intake characteristic according to EcoRI genotype.

	EcoRI polymorphism		
	GG	GA,AA	*P *value
Age (year)	53.82 ± 6.68	54.63 ± 6.24	0.17
Weight, Kg	75.57 ± 13.47	78 ± 14.34	0.06
Height, cm	161.37 ± 9.11	160.54 ± 8.56	0.07
BMI (kg/m2)	28.99 ± 4.47	30.25 ± 4.99	**0.004**
WC (cm)	91.50 ± 10.27	94.39 ± 11.78	**0.004**
HDL (mg/dl)	52.96 ± 11.64	55.56 ± 15.73	**0.03**
LDL (mg/dl)	108.49 ± 36.45	108.14 ± 33.04	0.91
LDL HDL	2.43 ± 7.68	3.30 ± 15.34	0.35
Cholesterol (mg/dl)	201.06 ± 75.43	202.87 ± 81.19	0.8
TG (mg/dl)	192.10 ± 113	183.04 ± 93.76	0.38
TG/HDL	3.78 ± 2.29	3.52 ± 2.17	0.22
Leptin (ng/ml)	24.97 ± 14.61	25.98 ± 14.78	0.67
Ghrelin (ng/ml)	2.05 ± 1.12	2.42 ± 1.20	0.06
CRP (mg/l)	2.25 ± 1.53	2.55 ± 1.44	0.32
IL-18(pg/ml)	247.43 ± 32.39	252.61 ± 25.61	0.4
Total energy intake, kcal/day	2595.30 ± 865.64	2526.01 ± 1051.63	0.42
Carbohydrate, g/day	341.70 ± 121.04	333.36 ± 157.19	0.49
Protein, g/day	90.33 ± 30.76	87.33 ± 41.04	0.33
Total fat, g/day	105.13 ± 48.51	100.36 ± 47.10	0.29
Saturated fatty acids, g/day	26.56 ± 10.39	26.87 ± 13.43	0.76
Monounsaturated fatty acids, g/day	36.12 ± 18.32	34.57 ± 18.06	0.36
Polyunsaturated fatty acids, g/day	26.50 ± 15.71	23.69 ± 12.43	0.02
Cholesterol, (mg/day)	227.80 ± 224.88	210.38 ± 119.23	0.21
Dietary fiber, g/day	42.18 ± 18.81	40.03 ± 19.93	0.25

Data are presented as mean ± standard deviation (SD).

*BMI* body mass index,* HDL-c* high density lipoprotein cholesterol, *LDL-c* low density lipoprotein cholesterol, *TG* triglyceride, *CRP* C-reactive protein, *IL18* interleukin 18, *PGF2α * prostaglandinF2α a. *P* values obtained from Independent T-test.

**Figure 1:**
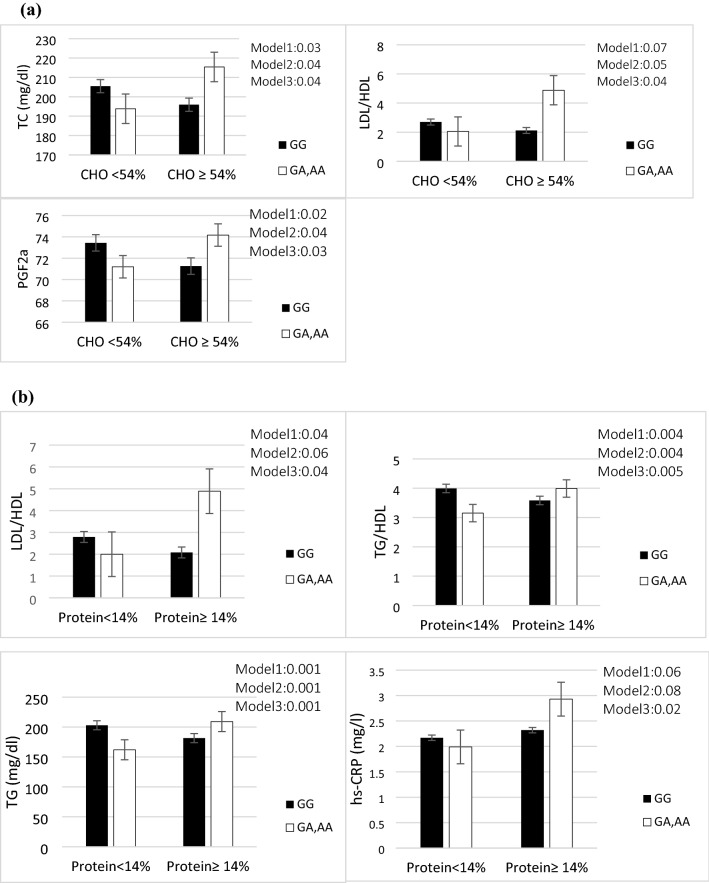

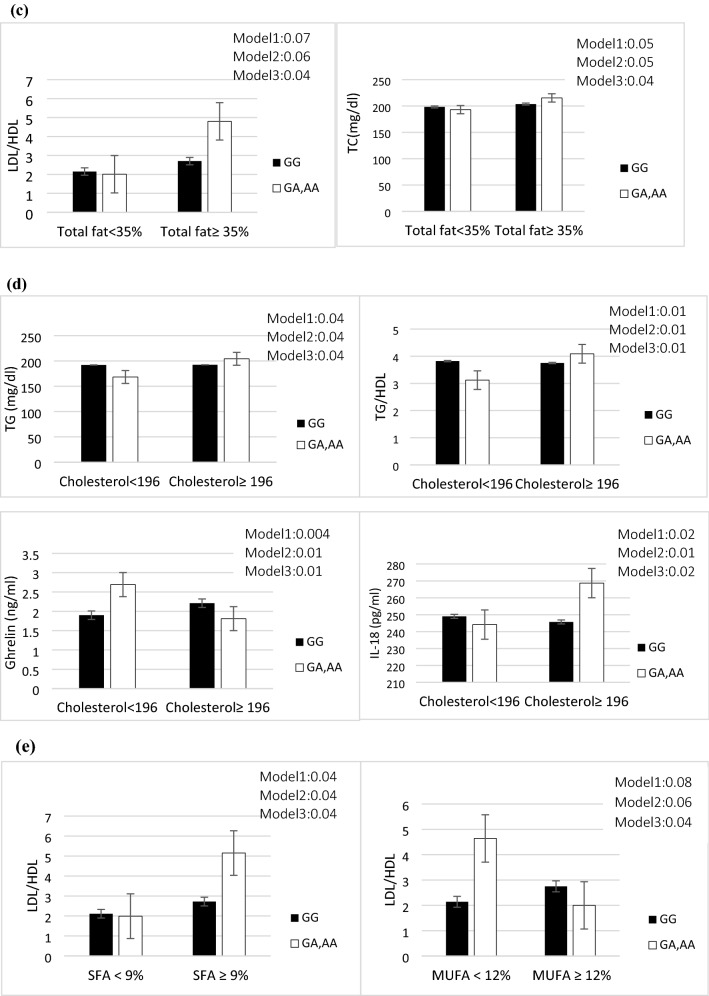
(**a**) Interaction between the ApoB ECORI and dietary carbohydrate intakes on serum TC, PGF2a level and LDL/HDL rati. *P* values for the interaction obtained in multivariate models using GLMs. Model 1: unadjusted; Model 2: adjusted for age, gender, physical activity, alcohol consumption and lipid-lowering medications; Model 3: adjusted for variables in model 2 plus for smoking, total energy intakes and fiber intake. Error bar: SEM. (**b**) Interaction between the ApoB ECORI and dietary protein intakes on serum TG, hs-CRP level, LDL/HDL and TG/HDL ratio. *P* values for the interaction obtained in multivariate models using GLMs. Model 1: unadjusted; Model 2: adjusted for age, gender, physical activity, alcohol consumption and lipid-lowering medications; model 3: adjusted for variables in model 2 plus for smoking, total energy intakes and fiber intake. Error bar: SEM. (**c**) Interaction between the ApoB ECORI and total fat intakes on serum TC level and LDL/HDL ratio. *P* values for the interaction obtained in multivariate models using GLMs. Model 1: unadjusted; Model 2: adjusted for age, gender, physical activity, alcohol consumption and lipid-lowering medications; Model 3: adjusted for variables in model 2 plus for smoking, total energy intakes and fiber intake. Error bar: SEM. (**d**) Interaction between the ApoB ECORI and cholesterol intakes on serum TG, ghrelin, IL-18 and level and TG/HDL ratio. *P* values for the interaction obtained in multivariate models using GLMs. Model 1: unadjusted; Model 2: adjusted for age, gender, physical activity, alcohol consumption and lipid-lowering medications; Model 3: adjusted for variables in model 2 plus for smoking, total energy intakes and fiber intake. Error bar: SEM. (**e**) Interaction between the ApoB ECORI and dietary specific fatty acids intakes containing SFA and MUFA intake on LDL/HDL ratio. *P* values for the interaction obtained in multivariate models using GLMs. Model 1: unadjusted; Model 2: adjusted for age, gender, physical activity, alcohol consumption and lipid-lowering medications; Model 3: adjusted for variables in model 2 plus for smoking, total energy intakes and fiber intake. Error bar: SEM.

The interactions between our genotype categories (GA, AA/ GG) and median value of total energy intake (54% for CHO, 14% for protein, 35% for total fat, 9% for SFA and 12% for MUFA) and 196 mg for cholesterol on lipid profiles and inflammatory markers are shown in Tables 3 and 4, respectively. Significant results are shown in Fig. 1a–e.

**Table 3:** Interactions of macronutrients intakes and ApoB ***EcoRI*** genotype on lipid profiles.

	Carbohydrate		Protein		Total fat		Cholesterol		SFA		MUFA	
	< 54%	≥ 54%	< 14%	≥ 14%	< 35%	≥ 35	< 196	≥ 196	< 9%	≥ 9%	< 12%	≥ 12%
	β (95%CI) *P*_interaction_		β (95%CI) *P*_interaction_		β (95%CI) *P*_interaction_		β (95%CI) *P*_interaction_		β (95%CI) *P*_interaction_		β (95%CI) *P*_interaction_	
LDL (mg/dl)												
GG (500)	107.78 ± 2.17	108.90 ± 2.32	109.41 ± 2.24	107.19 ± 2.24	109.07 ± 2.27	107.58 ± 2.21	106.02 ± 2.26	110.50 ± 2.22	110.60 ± 2.32	106.29 ± 2.17	109.19 ± 2.20	107.34 ± 2.28
GA,AA (143)	108.88 ± 3.98	107.18 ± 4.42	106 ± 4.07	110.67 ± 4.38	108.65 ± 4.32	107.65 ± 4.06	108.37 ± 3.83	107.81 ± 4.60	110.36 ± 4.56	106.50 ± 3.88	110.19 ± 4.20	106.08 ± 4.17
Model 1	− 2.82 (− 16.06, 10.41)0.67		6.89 (− 8.44, 4) 0.30		0.49 (− 12.7, 13.68) 0.94		− 5.03 (− 18.31, 8.24) 0.45		0.45 (− 12.83, 13.75) 0.94		− 2.26 (− 15.43, 10.90) 0.73	
Model 2	− 2.7 (− 15.91, 10.51) 0.68		6.5(− 6.68, 19.68) 0.33		0.49 (− 12.64, 13.62) 0.94		− 5.20 (− 18.42, 8.01) 0.44		− 0.10 (− 13.32, 13.12) 0.98		− 2.57 (− 15.68, 10.52) 0.70	
Model 3	− 2.84 (− 16.05, 10.36) 0.67		6.93 (− 6.27, 20.14) 0.30		0.41 (− 12.72, 13.55) 0.95		− 4.51 (− 17.76, 8.74) 0.50		− 0.94 (− 14.16, 12.27) 0.88		− 2.69 (− 15.8, 10.41) 0.68	
HDL (mg/dl)												
GG (500)	53.40 ± 0.77	52.34 ± 0.82	53.20 ± 0.79	52.61 ± 0.79	51.73 ± 0.80	54.01 ± 0.78	52.46 ± 0.80	53.32 ± 0.79	53.26 ± 0.82	52.58 ± 0.77	52.10 ± 0.78	53.77 ± 0.81
GA,AA (143)	55.67 ± 1.41	54.98 ± 1.57	55.79 ± 1.42	54.84 ± 1.56	54.61 ± 1.53	56.02 ± 1.44	56.50 ± 1.36	54.22 ± 1.64	54.33 ± 1.62	56.10 ± 1.38	54.63 ± 1.49	56.08 ± 1.48
Model 1	0.37 (− 4.33, 5.07) 0.87		− 0.35 (− 5.05, 4.34) 0.88		− 0.85 (− 5.53, 3.82) 0.71		− 3.14 (− 7.89, 1.59) 0.19		2.45 (− 2.27, 7.18) 0.31		− 0.22 (− 4.90, 4.45) 0.92	
Model 2	− 0.52 (− 5.11, 4.07) 0.82		0.23 (− 4.37, 4.84) 0.92		− 0.47 (− 5.05, 4.11) 0.84		− 3.25 (− 7.87,1.37) 0.16		2.73 (− 1.88, 7.34) 0.24		− 0.19 (− 4.75, 4.37) 0.93	
Model 3	− 0.67 (− 5.26, 3.91) 0.77		0.53 (− 4.06,5.12) 0.82		− 0.34 (− 4.91, 4.22) 0.88		− 2.85 (− 7.46, 1.75) 0.22		2.71 (− 1.90, 7.32) 0.25		− 0.15 (− 4.71, 4.40) 0.94	
LDL/HDL												
GG (500)	2.70 ± 0.60	2.12 ± 0.64	2.79 ± 0.62	2.08 ± 0.62	2.15 ± 0.63	2.70 ± 0.61	2.77 ± 0.63	2.11 ± 0.62	2.11 ± 0.64	2.72 ± 0.60	2.14 ± 0.61	2.75 ± 0.64
GA,AA (143)	2.05 ± 1.11	4.88 ± 1.23	2 ± 1.12	4.89 ± 1.22	2.01 ± 1.13	4.80 ± 1.20	2.04 ± 1.28	4.17 ± 1.07	1.99 ± 1.08	5.15 ± 1.27	4.64 ± 1.17	2 ± 1.16
Model 1	3.41 (− 0.28, 7.11) 0.07		0.07 (0.003, 0.13) **0.04**		3.33 (− 0.35, 7.02) 0.07		− 1.47 (− 5.19, 2.25) 0.40		3.77 (0.05, 7.49) **0.04**		− 3.25 (− 6.93, 0.43) 0.08	
Model 2	3.59 (− 0.1, 7.3) 0.05		3.43 (− 0.26, 7.13) 0.06		3.42 (− 0.26, 7.1,) 0.06		− 1.36 (− 5.08, 2.35) 0.47		3.88 (0.16, 7.59,) **0.04**		− 0.05 (− 0.10, 0.004) 0.06	
Model 3	3.72 (0.01, 7.42) **0.04**		3.65 (0.04, 7.36) **0.04**		3.58 (0.10, 7.27) **0.04**		− 1.30 (− 5.03, 2.43) 0.49		3.74 (0.03, 7.46) **0.04**		− 0.44 (− 0.88, − 0.003) **0.04**	
TC (mg/dl)												
GG (500)	205.47 ± 4.68	195.90 ± 5.01	206.53 ± 4.86	195.55 ± 4.83	198.26 ± 4.91	203.61 ± 4.78	197.37 ± 4.87	204.57 ± 4.82	196.05 ± 5.01	205.39 ± 4.70	196.74 ± 4.76	205.58 ± 4.93
GA,AA (143)	193.82 ± 8.58	215.37 ± 9.54	201.82 ± 8.65	205.44 ± 9.48	193.05 ± 8.76	215.28 ± 9.33	196.16 ± 8.29	212.54 ± 9.95	212.01 ± 9.86	197.28 ± 8.39	211.77 ± 9.07	195.27 ± 9.01
Model 1	31.12 (2.58, 59.65) **0.03**		14.61 (− 13.92, 42.14) 0.31		27.57 (− 0.89, 56.04) 0.05		9.18 (− 19.55, 37.92) 0.53		− 24.06 (− 52.8, 4.67) 0.10		− 25.33 (− 53.77, 3.09) 0.08	
Model 2	29.95 (1.37, 58.53) **0.04**		15.43 (− 13.14, 44.01) 0.29		27.5 (− 0.93, 55.94) 0.05		9.23 (− 19.46, 37.93) 0.52		− 23.75 (− 52.45, 4.94) 0.10		− 3.30 (− 6.98, 0.38) 0.07	
Model 3	29.36 (0.87, 57.92) **0.04**		16.89 (− 11.68, 45.47) 0.24		27.02 (1.36, 55.40) **0.04**		9.02 (− 19.70, 37.74) 0.53		− 22.65 (− 51.34, 6.03) 0.12		− 25.85 (− 54.22, 2.50) 0.06	
TG (mg/dl)												
GG (500)	191.54 ± 6.71	192.88 ± 7.26	202.96 ± 6.94	181.54 ± 6.88	188.39 ± 7.07	195.72 ± 6.87	192.03 ± 7.04	192.28 ± 6.86	192.89 ± 7.23	191.52 ± 6.74	189.88 ± 6.87	194.58 ± 7.07
GA,AA (143)	190.48 ± 12.25	174.22 ± 13.72	162.02 ± 12.23	209.15 ± 13.50	185.25 ± 13.41	181.53 ± 12.49	168.22 ± 11.85	204.15 ± 14.13	187.61 ± 14.19	180.18 ± 11.96	190.55 ± 13.02	176.18 ± 12.83
Model 1	− 17.6 (− 58.55, 23.34) 0.39		68.55 (28.02, 109.08) **0.001**		− 11.04 (− 51.85, 29.76) 0.59		0.08 (0.001, 0.17) **0.04**		− 6.05 (− 47.27, 35.16) 0.77		− 19.07 (− 59.80, 21.64) 0.35	
Model 2	− 18.33 (− 59.15, 22.48) 0.37		70.15 (29.68, 110.62) **0.001**		− 12.73 (− 53.31, 27.84) 0.53		0.09 (0.003, 0.17) **0.04**		− 5.36 (− 46.36, 35.62) 0.79		− 18.85 (− 59.35, 21.63) 0.36	
Model 3	− 19.8 (− 60.54, 20.92) 0.34		69.59 (29.16, 110.03) **0.001**		− 9.80 (− 50.35, 30.74) 0.63		0.19 (0.02, 0.36) **0.02**		− 3.58 (− 44.54, 37.38) 0.86		− 18.01 (− 58.45, 22.42) 0.38	
TC/HDL												
GG (500)	3.01 ± 0.10	2.90 ± 0.11	3.06 ± 0.11	2.87 ± 0.11	2.99 ± 0.11	2.94 ± 0.11	2.93 ± 0.11	2.99 ± 0.11	2.84 ± 0.11	3.07 ± 0.10	2.92 ± 0.11	3.01 ± 0.11
GA,AA (143)	2.75 ± 0.20	3.15 ± 0.22	2.90 ± 0.20	2.97 ± 0.22	3.20 ± 0.21	2.69 ± 0.20	2.66 ± 0.19	3.27 ± 0.23	3.18 ± 0.22	2.75 ± 0.19	3.06 ± 0.21	2.80 ± 0.20
Model 1	0.51 (− 0.15, 1.17) 0.13		0.25 (− 0.41, 0.91) 0.45		− 0.46 (− 1.12, 0.20) 0.17		0.55 (− 0.11, 1.22) 0.10		− 0.64 (− 1.31, 0.02) 0.05		− 0.33 (− 0.99, 0.33) 0.32	
Model 2	0.56 (− 0.09, 1.22) 0.09		0.22 (− 0.43, 0.89) 0.49		− 0.50 (− 1.16, 0.15) 0.13		0.55 (− 0.10, 1.22) 0.10		− 0.65 (− 1.32, 0.009) 0.05		− 0.34 (− 1.005, 0.31) 0.30	
Model 3	0.55 (− 0.1, 1.22) 0.09		0.24 (− 0.41, 0.90) 0.47		− 0.49 (− 1.14, 0.17) 0.14		0.53 (− 0.12, 1.20) 0.11		− 0.62 (− 1.28, 0.04) 0.06		− 0.32 (− 1.01, 0.30) 0.29	
TG/HDL												
GG (500)	3.78 ± 0.14	3.79 ± 0.15	3.99 ± 0.14	3.58 ± 0.14	3.76 ± 0.14	3.81 ± 0.14	3.82 ± 0.14	3.75 ± 0.14	3.76 ± 0.15	3.81 ± 0.14	3.75 ± 0.14	3.82 ± 0.14
GA,AA (143)	3.65 ± 0.25	3.79 ± 0.15	3.15 ± 0.25	3.99 ± 0.14	3.60 ± 0.27	3.47 ± 0.26	3.12 ± 0.24	4.09 ± 0.29	3.71 ± 0.29	3.40 ± 0.24	3.64 ± 0.27	3.42 ± 0.26
Model 1	− 0.29 (− 1.15, 0.55) 0.49		1.26 (0.41, 2.108) **0.004**		− 0.17 (− 1.02, 0.68) 0.69		1.04 (0.19, 1.89) **0.01**		− 0.35 (− 1.21, 0.50) 0.41		− 0.28 (− 1.13, 0.56) 0.50	
Model 2	− 0.24 (− 1.08, 0.6) 0.57		1.24 (0.4, 2.08) **0.004**		− 0.24 (− 1.08, 0.59) 0.56		1.07 (0.23, 1.91) **0.01**		− 0.35 (− 1.19, 0.49) 0.41		− 0.28 (− 1.12, 0.54) 0.49	
Model 3	− 0.26 (− 1.1, 0.57)0.54		1.20 (0.37, 2.04) **0.005**		− 0.18n (− 1.01, 0.65) 0.66		1.02 (0.18, 1.87) **0.01**		− 0.31 (− 1.15, 0.53) 0.46		− 0.27 (− 1.10, 0.56) 0.52	

*CI* confidence interval, *TC* total cholesterol, *TG* triglyceride, *HDL* high density lipoprotein, *LDL* low density lipoprotein.

GG genotype is considered as a reference. Low median intakes of macronutrients considered as a reference.

*P* values for the interaction obtained in multivariate models using GLMs. Model 1: unadjusted; Model 2: adjusted for age, gender, physical activity, alcohol consumption and lipid-lowering medications; model 3: adjusted for variables in model 2 plus for smoking, total energy intakes and fiber intake.

**Table 4:** Interactions of macronutrients intakes and ApoB ***EcoRI*** genotype on leptin, ghrelin and inflammatory markers.

	Carbohydrate		Protein		Total fat		Cholesterol		SFA		MUFA	
	< 54%	≥ 54%	< 14%	≥ 14%	< 35%	≥ 35	< 196	≥ 196	< 9%	≥ 9%	< 12%	≥ 12%
	β (95%CI) *P*_interaction_		β (95%CI) *P*_interaction_		β (95%CI) *P*_interaction_		β (95%CI) *P*_interaction_		β (95%CI) *P*_interaction_		β (95%CI) *P*_interaction_	
Leptin												
GG (257)	27.13 ± 1.54	22.34 ± 1.70	25.51 ± 1.64	24.43 ± 1.63	23.46 ± 1.67	26.30 ± 1.58	24.23 ± 1.64	25.69 ± 1.63	25.42 ± 1.61	24.50 ± 1.64	24.11 ± 1.53	26.09 ± 1.75
GA,AA (147)	27.26 ± 2.93	25.67 ± 2.99	27.64 ± 2.96	25.27 ± 3.02	25.02 ± 2.88	28.14 ± 3.08	25.56 ± 2.61	26.74 ± 3.53	23.71 ± 3.32	28.36 ± 2.73	24.01 ± 2.88	29.29 ± 3.07
Model 1	3.2 (− 6.17, 12.57) 0.50		− 1.28 (− 10.73, 8.16) 0.79		0.27 (− 9.16, 9.7) 0.95		− 0.28 (− 10.03, 9.45) 0.95		5.57 (− 4, 15.14) 0.25		3.29 (− 6.15, 12.73) 0.49	
Model 2	4.01 (− 4.77, 12.81) 0.37		1.47 (− 7.39, 10.35) 0.74		− 1.95 (− 10.78, 6.87) 0.66		2.41 (− 6.61, 11.44) 0.60		2.97 (− 5.93, 11.87) 0.51		0.79 (− 8.04, 9.63) 0.86	
Model 3	3.83 (− 4.93,12.59) 0.39		1.22 (− 7.62, 10.06) 0.78		− 3.31 (− 12.1, 5.48) 0.46		1.02 (− 8.22, 10.27) 0.82		2.76 (− 6.12, 11.64) 0.54		0.69 (− 8.12, 9.51) 0.87	
Ghrelin												
GG (257)	2 ± 0.12	2.12 ± 0.13	1.98 ± 0.13	2.12 ± 0.12	2.17 ± 0.12	1.94 ± 0.13	1.90 ± 0.12	2.21 ± 0.12	2.22 ± 0.12	1.86 ± 0.13	2.09 ± 0.11	2.01 ± 0.14
GA,AA (147)	2.30 ± 0.24	2.54 ± 0.24	2.15 ± 0.24	2.72 ± 0.25	2.61 ± 0.23	2.18 ± 0.25	1.81 ± 0.30	2.69 ± 0.20	2.62 ± 0.26	2.27 ± 0.22	2.73 ± 0.23	2.04 ± 0.25
Model 1	0.12 (− 0.65, 0.89) 0.75		0.43 (− 0.33, 1.20) 0.27		− 0.19 (− 0.97, 0.57) 0.61		1.19 (0.39, 2) **0.004**		0.009 (− 0.76, 0.78)0.98		− 0.6 (− 1.37, 0.17) 0.12	
Model 2	0.09 (− 0.67, 0.86) 0.81		0.56 (− 0.19, 1.32) 0.14		− 0.04 (− 0.09, 0.01) 0.14		1.04 (0.24, 1.85) **0.01**		0.03 (− 0.72, 0.79) 0.93		− 0.14 (0.29, 0.006) 0.06	
Model 3	0.18 (− 0.57, 0.94) 0.62		0.55 (− 0.19, 1.30) 0.14		− 0.31 (− 1.07, 0.43) 0.41		1.02 (0.21, 1.82) **0.01**		0.03 (− 0.7, 0.78) 0.91		− 0.68 (− 1.43, 0.06) 0.05	
CRP												
GG (257)	2.19 ± 0.19	2.32 ± 0.20	2.17 ± 0.20	2.32 ± 0.18	2.38 ± 0.19	2.10 ± 0.20	2.25 ± 0.19	2.25 ± 0.20	2.28 ± 0.20	2.23 ± 0.18	2.39 ± 0.18	2.08 ± 0.20
GA,AA (147)	2.74 ± 0.38	2.38 ± 0.36	1.99 ± 0.41	2.93 ± 0.34	2.42 ± 0.37	2.68 ± 0.37	2.38 ± 0.32	2.87 ± 0.45	2.26 ± 0.43	2.72 ± 0.33	2.28 ± 0.36	2.85 ± 0.38
Model 1	− 0.49 (− 1.67, 0.68) 0.40		1.1(− 0.08, 2.28) 0.06		0.53 (− 0.63, 1.71) 0.36		0.48 (− 0.74, 0.58) 0.44		0.50 (− 0.69, 1.71) 0.40		0.88 (− 0.28, 2.05) 0.14	
Model 2	− 0.54 (− 1.73, 0.64) 0.36		1.05 (− 0.14, 2.24) 0.08		0.53 (− 0.64, 1.72) 0.37		0.64 (− 0.59, 1.88) 0.30		0.63 (− 0.57, 1.84) 0.30		0.89 (− 0.27, 2.07) 0.13	
Model 3	− 0.53 (− 1.7, 0.63) 0.37		1.32 (0.13, 2.51,) **0.02**		0.72 (− 0.44, 1.89) 0.22		0.74 (− 0.46,1.94) 0.23		0.64 (− 0.52, 1.82) 0.28		0.97 (− 0.18, 2.12) 0.10	
IL− 18												
GG (257)	254.96 ± 3.84	239.09 ± 4.04	248.41 ± 4.19	246.59 ± 3.91	238.35 ± 3.79	257.51 ± 3.99	249.03 ± 3.91	245.72 ± 4.05	240.94 ± 4.21	252.71 ± 3.80	239.04 ± 3.71	257.76 ± 4.12
GA,AA (147)	255.44 ± 7.75	250.12 ± 7.28	252.78 ± 7.07	252.38 ± 8.55	249.73 ± 7.41	255.50 ± 7.41	244.18 ± 6.62	268.71 ± 9.15	253.34 ± 8.78	252.17 ± 6.80	249.60 ± 7.20	256.03 ± 7.67
Model 1	10.55 (− 12.99, 34.10) 0.38		1.41 (− 23.09, 25.92) 0.91		− 13.39 (− 36.60, 9.82) 0.25		27.84 (3.09, 52.59) **0.02**		− 12.93 (− 37.39,….) 0.30		− 12.29 (− 35.60, 11.02) 0.30	
Model 2	11.14 (− 12.73, 35.01) 0.36		0.79 (− 24.02, 25.60) 0.95		− 14.27 (− 37.73, 9.19) 0.23		30.89 (5.74, 56.05) **0.01**		− 12.73 (− 37.5, 12.04) 0.31		− 12.28 (− 35.76, 11.19) 0.30	
Model 3	10.6 (− 13.49, 34.70) 0.38		1.35 (− 24.01, 26.73) 0.91		− 13.24 (− 37.1, 10.61) 0.27		29.59 (4.53, 54.65) **0.02**		− 13.97 (− 38.66, 10.71) 0.26		− 11.84 (− 35.52, 11.83) 0.32	
PGF2a												
GG (257)	73.44 ± 0.75	71.26 ± 0.78	73.11 ± 0.80	71.77 ± 0.75	71.75 ± 0.76	73.10 ± 0.79	72.45 ± 0.77	73.19 ± 1.29	72.43 ± 0.82	72.37 ± 0.74	71.66 ± 0.73	73.32 ± 0.82
GA,AA (147)	71.20 ± 1.50	74.17 ± 1.41	72.05 ± 1.35	73.84 ± 1.63	73.56 ± 1.47	72 ± 1.47	72.33 ± 0.79	72 ± 1.78	73.33 ± 1.71	72.45 ± 1.32	73.82 ± 1.42	71.60 ± 1.51
Model 1	5.15 (0.59, 9.71) **0.02**		3.13 (− 1.54, 7.81) 0.18		− 2.90 (− 7.52, 1.71) 0.21		− 1.07 (− 5.9, 3.76) 0.66		− 0.82 (− 5.59, 3.94) 0.73		− 3.88 (− 8.49, 0.72) 0.09	
Model 2	4.74 (0.2, 9.29) **0.04**		2.56 (− 2.07, 7.19) 0.27		− 2.4 (− 6.99, 2.17) 0.30		− .66 (− 5.48, 4.15) 0.78		− 0.66 (− 5.37,4.05) 0.78		− 3.44 (− 8, 1.11) 0.13	
Model 3	5.01 (0.48, 9.53) **0.03**		2.57 (− 2.15, 7.31)0.28		− 2.16 (− 6.8, 2.46) 0.35		− 0.82 (− 5.59, 3.94) 0.73		− 0.91 (− 5.58, 3.74) 0.70		− 3.55 (− 8.09, 0.98) 0.12	

*CRP* C-reactive protein, *IL18* interleukin 18, *PGF2α* prostaglandinF2α.

GG genotype is considered as a reference. Low median intakes of macronutrients considered as a reference.

*P* values for the interaction obtained in multivariate models using GLMs. Model 1: unadjusted; Model 2: adjusted for age, gender, physical activity, alcohol consumption and lipid-lowering medications; model 3: adjusted for variables in model 2 plus for smoking, total energy intakes and fiber intake.

According to our findings on the gene-diet interaction, ApoB EcoRI polymorphism and CHO intake had a significant interaction on serum TC (*P* = 0.03) and PGF2a level (*P* = 0.03) in three models. TC and PGF2a concentrations were greatly increased in patients with (AA or AG) genotypes with higher than median of CHO intake (≥ 54% of total energy) compared to the GG genotype. Besides, we observed significant results on LDL/HDL ratio after adjustment for potential confounders in two models (*P* = 0.04). Therefore, A-allele carriers with higher than median of CHO intake have a higher LDL/HDL ratio compared to GG homozygotes (Fig. 1a).

Also, we revealed that A-allele carriers who consumed a more than median of protein diet (≥ 14 percent of total energy) had greater TG and CRP levels in their plasma, as well as a higher TG/HDL and LDL/HDL ratio, compared to GG genotypes (*P* = 0.001, *P* = 0.02, *P* = 0.005 and *P* = 0.04 respectively) (Fig. 1b). Moreover, other significant results were detected between ApoB EcoRI and total fat intake on TC level and LDL/HDL ratio in the adjustment model (model 3) (*P* = 0.04 and *P* = 0.04 respectively). The A-allele carriers with higher than median intake of total fat (≥ 35% of total energy) revealed significantly increased serum TC level and LDL/HDL ratio compared to GG homozygotes (Fig. 1c).

In crude and two adjusted models, we found a significant interaction between the ApoB EcoRI polymorphism and cholesterol intake on TG, TG/HDL ratio, and IL-18. Therefore, A-allele carriers with above than median cholesterol intake (> 196 mg) have higher TG, TG/HDL ratio, and IL-18 compared to GG homozygotes (*P* = 0.04, *P* = 0.01, and *P* = 0.02 respectively).

Dietary components such as total fat and type of dietary fat, as well as HOMA-IR, are positively related to serum leptin concentrations while serum ghrelin concentrations are negatively related^30^. In the present study, diabetic patients with GA, AA genotype who consumed higher than median cholesterol have lower ghrelin concentration (*P* = 0.01) (Fig. 1d).

Finally, we found a considerable interaction between the ApoB EcoRI variant and various fatty acid intakes (≥ 9 percent for SFA, ≥ 12 percent for MUFA) on the LDL/HDL ratio. A-allele carriers who consume above than median of SFA intake have a higher LDL/HDL ratio (*P* = 0.04), but this relationship is inverted (*P* = 0.04) when they consume higher than median median of MUFA (Fig. 1e).

## Discussion

The ApoB EcoRI polymorphism was found to have a significant relationship with serum HDL-C levels and obesity indices in the current work. A-allele carriers are more sensitive to dyslipidemia and CVD than GG homozygotes because they have a considerably higher general obesity risk than GG genotype^17^. However, HDL concentration was higher between A –allele carriers than GG genotype. These findings are inconsistent with other study has shown participant with A/A genotype reported considerably greater HDL-C and apoA1 levels, as well as decreased total protein and albumin levels in their plasma compared with GA or GG carriers, however no significant differences in BMI was found^17^. Based on previous studies, amino acid 4154 is located in the apoB protein's C-terminal domain, which is essential for lipid association. This polymorphism induced amino acid substitution has the potential to have a significant impact on protein function and, as a result, lipid levels^31^. Our hypothesis is supported by the findings of this investigation, however, it contradicts the findings of a previous study which revealed that no influence of the EcoRI polymorphism on serum lipid levels in healthy subjects^32^.

Analyzing the interactions between ApoB EcoRI polymorphism and macronutrient intake on lipid profiles showed significant interaction. A-allele carriers with above than median dietary CHO, protein, total fat and SFA intake showed a significantly higher serum LDL/HDL ratio compared to GG homozygotes, also when they consumed higher than median of MUFA this association was inverse. Moreover, we observed that A-allele carriers with a above than median of CHO and total fat had considerably greater serum levels of TC than GG genotype carriers. Furthermore, when protein intake was ≥ 14% of total energy or cholesterol intake more than 196 mg, we observe a significantly higher TG serum level and TG/HDL ratio between A-allele carriers compared to GG genotype. The EcoRI polymorphism influenced the response of plasma TC, LDL, TG, LDL/HDL, and TG/HDL ratios to diet in the current investigation. When AA, AG patients were shifted from a below to above median of fat inrake, or from a below to above median of cholesterol diet, their plasma lipids increased the most. In particular, we found a unique interaction between the ApoB EcoRI polymorphism and macronutrient intake on inflammatory markers and appetite-related hormones in the current study. When cholesterol intake was higher than median intake, patients with AG and AA genotypes displayed significantly higher ghrelin and IL-18, also when consumed than median protein intake they have shown higher CRP. On the other hand, above the median of CHO intake, carriers of A allele were associated with higher PGF2a serum levels than GG genotype.

According to these findings, higher than median of MUFA consumption may reduce the EcoRI rs1042031 associations with cardiometabolic markers with a lower LDL/HDL ratio, but individuals with a higher genetic susceptibility seemed to have an increased chance of cardiometabolic disease, which was related to more than median dietary CHO, protein, total fat, SFA, and cholesterol consumption. Thus, considering adherence to a diet rich in CHO, protein, total fat, SFA and cholesterol can be expected to see the more atherogenic effect in subjects with the AA OR AG genotype.

To the best of our knowledge, this is the first study that shows interaction between dietary intake and ApoB EcoRI on dyslipidemia, inflammatory markers and appetite-related hormones. The EcoRI allele was shown to be more common in the responders than the non-responders between fifty-one participants who were classed as different dietary intakes^33^. Gylling et al. evaluated the effect of ApoB EcoRI and food on lipid profile in a study similar to ours. In comparison to patients with the GG genotype, carriers of the ApoB EcoRI R + (A) allele showed the most pronounced LDL cholesterol increase and fractional metabolism rate for LDL when shifting from low to high cholesterol and SFA intake^34^ In line with our findings, Rantala et al. showed that AA individuals had significantly higher total and LDL-cholesterol responses than GG patients in a meta-analysis of seven investigations. Apo-B EcoRI Polymorphisms is responsiveness to diet high in fat contains (45 percent of energy was derived from CHO, 18 percent from protein, and 36 percent from fat and daily cholesterol intake was 420 mg) which is induced a greater increase LDL level in patients carrying the AA genotype compared in someone with the GA or GG genotype, but no significant correlation was found in the low-fat low-cholesterol diet^26^. Furthermore, Friedlander et al. found that when participants heterozygous for the less common apo B EcoRI allele (AG) consumed a high dietary fat intake, plasma cholesterol and LDL-C levels changed significantly compared to homozygous carriers^35^. However, some studies have shown no association between EcoRI polymorphism and dietary intake. According to a recent study, a higher intake of wheat- or oat-bran supplementation as dietary fiber did not affect plasma apolipoprotein B-containing lipoprotein concentrations when the ApoB EcoRI polymorphismon was examined^36^. Another prospective double-blind crossover dietary study found that EcoRI has not fully responded to dietary fat intake. Dietary participants were randomized to drink either a fat- and cholesterol-free liquid supplement or one that contained fat (30–36 g) and cholesterol for three weeks (650–780 mg)^37^. Friedlander et al. also found no evidence of an association between dietary (low-SFA and high-PUFA) and the apoB EcoRI polymorphism on metabolic indicators^38^. Except for PUFA intake in our study, other interactions identified for CHO, protein, total fat, SFA, MUFA, and cholesterol of diet on dyslipidemia, inflammatory markers, and appetite-related hormones.

The EcoRI polymorphism's biological function is unclear, hence the mechanism by which it alters metabolic indicators in response to dietary intake is unknown. Variations in the synthesis rate or apo B and all lipoproteins containing apo B catabolism with variable food intake might explain this connection. Previous research has found it difficult to build conclusions about the significance of this polymorphism for cholesterol metabolism since the results are few and contradictory, ranging from no association with cholesterol levels to a strong correlation^39–46^ to some association with other lipid markers includes TG and VLDL levels^40,46–49^. This polymorphism is caused by a single base-pair mutation in the apo B gene's coding area (exon 29), which modifies the amino acid sequence and makes a functional role^50^. Furthermore, because this polymorphism is found in ApoB's carboxy-terminal tail, it may impact ApoB's ability to connect to LDL receptors^51^. It's conceivable that this amino acid alteration affects the encoded apo B molecules' LDL receptor affinity^45^. The apo B EcoRI polymorphism was also connected to cholesterol absorption efficiency in a previous investigation. Cholesterol production and LDL receptor function would be significantly suppressed when cholesterol uptake capacity is high, as it is in the R + patients^52–54^. These findings suggest that common polymorphism of the apo B EcoRI could cause various responses in plasma TC and LDL levels when dietary cholesterol content is significantly changed. It seems these modification was done by changing apo B secretion, LDL receptor affinity and structural stability or combinations of lipoproteins containing apo B with other lipoproteins or different kinds of enzymes such as LP^26^.

In this term, experimental studies have shown that compared to wild-type (WT) mice on normal diets, Apobec-1 knockout animals (KO) containing apoB100 but not apoB48 effectively make chylomicrons and can absorb dietary fat without obvious deficiency. The frequency of chylomicron secretion was influenced by the migration of apo-B-containing particles into the lumen of the smooth endoplasmic reticulum when dietary TG was low. The rate-limiting process appeared to be the insertion of TG to the apo-B-containing molecules when dietary TG is high (Western diet). In this regard, the findings indicate that apo-B100 construction in chylomicrons is inefficient in the early sTGes of chylomicron assembly in the KO mice, but that intracellular TG accumulation (due to a high-fat diet) surpasses this inefficiency and promotes chylomicron secretion at a higher level. This could also explain why chylomicrons from fat-fed KO mice are 66 percent larger than those from WT mice consuming the same diet. The accumulating TG in the enterocytes may result in bigger TG particles that can be assembled with apo-B100-containing precursors^55^. Therefore, Another possible pathway by which the EcoRI in this investigation could influence the association between dietary macronutrient intake and lipid profile is at the absorption or chylomicron assembly sTGes in the small intestine.

Furthermore, the amount of hepatic apoB release, and thus LDL density, might also be affected by the amount of fatty acid taken up by peripheral tissues. Some authors observed that decreased fatty acid uptake might lead to an increased source of hepatic TG. The resultant raised levels of fatty acids generated in the liver would lead to increased hepatic secretion of apoB containing lipoproteins^56^. Genest et al. backed up this hypothesis by experimental study, it has been demonstrated that, normotriglyceridaemic (NTG) hyper-apoB participants remove TGs more slowly following an oral fat load versus healthy subjects^57^, and that decreased fatty acid uptake by peripherial tissues leads to slower TG synthesis in NTG hyper-apoB individuals' adipocytes^58^.

Moreover, according to a previous study, decreased ApoB synthesis in the body leads to increased production of leptin and ghrelin, which leads to resistance and obesity^59^. Furthermore, leptin is responsive to dietary changes, particularly in diabetic patients^60^. Increased ghrelin levels are linked to lower HDL cholesterol levels and higher LDL cholesterol levels^61^. Dietary components such as total fat and type of dietary fat, as well as HOMA-IR, are positively related to serum leptin concentrations while serum ghrelin concentrations are negatively related^30^. BMI, nonHDL, and fibrinogen were found to be strong predictors of inflammatory marker concentrations in a multinomial logistic regression study^62^. Factors such as lipid metabolism, blood pressure, obesity, glucose metabolism, renal function, and lifestyle had varying effects on the development of the disease through inflammatory factors^63^.

Therefore, in this study, the pathway through which macronutrients affect the genetic risk provided by EcoRI polymorphisms is unknown; more research is needed to identify such gene-diet treatments in diabetic patients. The Apo B EcoRI polymorphism could be a positive figure for further information on how to manage metabolic disorders in T2DM patients.

### Limitations and strengths

Our study is the first study to examine the existence of Apo-B *EcoRI* gene interaction with macronutrient intake on lipid profile and inflammatory markers in T2DM patients to date. Our study was included all different types of dietary intake. We adjusted to wide range of confounders. Although we provide a novel addition to the literature, some limitations should be considered in the interpretation of this study. It is impossible to argue that our study's cross-sectional design generated any causality; the use of FFQ for dietary assessing, which may have resulted in memory bias. Furthermore, our participants were from the Iranian country which may not be generalized due to racial and regional differences. ApoB EcoRI mutations altered metabolic diseases that responded to dietary changes in our research sample. Although other components might also be implicated and should be investigated further in larger research to identify their probable impact in plasma lipid response to diet and so help to reveal the mechanisms behind differential responsiveness. This may be due to the interaction between unmeasured genetic variation or environmental factors that were different between the healthy individuals and diabetic patients.

## Conclusion

Our study showed that plasma lipid concentrations in subjects with the deletion allele (AA or AG) may also be more responsive to an increased percenTGe of energy from dietary fat, CHO, protein, SFA, and cholesterol consumption. Therefore, individuals with greater genetic predisposition (AA or AG) appeared to have higher metabolic markers with higher percenTGe of macronutrient consumption and higher MUFA consumption might attenuate the ApoB EcoRI associations with metabolic markers.

## Data Availability

The data are not publicly available due to containing private information of participants. Data are however available from the authors upon reasonable request and with permission of Fariba Koohdani.

## Abbreviations

T2DM: Type 2 diabetes mellitus
HDL: High-density lipoprotein cholesterol
LDL: Low-density lipoprotein cholesterol
VLDL: Very low-density lipoprotein
TG: Triglyceride
TC: Total cholesterol
SNP: Single nucleotide polymorphism
PCR–RFLP: Polymerase chain reaction–restriction fragment length polymorphism
GLM: Generalized linear model
BMI: Body mass index
WC: Waist circumference
